# Reply to letter to editor

**DOI:** 10.1007/s10194-012-0483-0

**Published:** 2012-09-13

**Authors:** Jennifer Sayanlar, Nilufer Guleyupoglu, Russell Portenoy, Sait Ashina

**Affiliations:** Department of Pain Medicine and Palliative Care, and Department of Neurology, Albert Einstein College of Medicine, Beth Israel Medical Center, New York, NY USA

We appreciate Dr. Kapoor’s interest in our article on the treatment of postherpetic neuralgia with the capsaicin 8 % patch [1]. Postherpetic neuralgia can be very difficult to treat and the development of new therapies is needed, particularly given the potential for side effects from current systemic therapies [2]. Vitamin C has low side effect liability and has been found to favorably affect one type of neuropathic pain, reducing the risk of complex regional pain syndrome following the wrist fracture [3]. We agree that further investigation of this agent in other types of neuropathic pain, such as postherpetic neuralgia, is warranted.
